# Lightweighting in the automotive industry as a measure for energy efficiency: Review of the main materials and methods

**DOI:** 10.1016/j.heliyon.2024.e29728

**Published:** 2024-04-16

**Authors:** Andrea Candela, Giulia Sandrini, Marco Gadola, Daniel Chindamo, Paolo Magri

**Affiliations:** Department of Mechanical and Industrial Engineering, University of Brescia, I-25123, Brescia, Italy

**Keywords:** Weight reduction, Fuel reduction value (FRV), Energy reduction value (ERV), Longitudinal dynamics simulation, Energy savings, Automotive sustainability

## Abstract

The increasing emissions of greenhouse gases (GHG) and pollutants like particulate matter and nitrogen oxides (NOx) have led to environmental concerns. Hybrid and electric powertrains are being introduced as means to reduce pollutant emissions, especially at the local level. Additionally, the finite availability of fossil fuel sources, which are used to produce gasoline and diesel, highlights the need for alternative technical solutions. One approach to partly address these issues is lightweighting, which involves reducing the weight of vehicles to minimize their impact during the use phase. Mathematical models are employed to simulate the longitudinal dynamics of vehicles and estimate the energy required to accomplish driving missions. Appropriate metrics have been developed to quantify energy-saving effects that, in addition, can support the decision making, design, and development phase of future vehicles. To facilitate this process, it would be useful to build a database of ERV (Energy Reduction Value) and FRV (Fuel Reduction Value) figures derived through a unified procedure. Such a database would be useful in evaluating the effectiveness of vehicle lightweighting and its impact on energy consumption and pollutant emissions. The last phase of the analysis is the assessment of the overall reduction in the environmental impact of the vehicle throughout its life cycle by using the LCA (Life Cycle Assessment) approach. From this study, it was possible to conclude that lightweighting can be an appropriate solution to improve the energy efficiency of vehicles and that appropriate metrics, can support the development of new car models. The potential to integrate enhanced energy efficiency, lower emissions, and higher safety features into our everyday vehicles would represent a significant advancement in the automotive industry. There is a gap in the scientific literature on the effects of lightweighting on vehicle dynamics and energy usage which deserves to be investigated.

## 1 Introduction

Nowadays, the issues of climate change [1] caused by excessive greenhouse gas emissions [2] is the main problem that humanity will have to deal with in the coming years. The depletion of fossil fuels is also a concern [3]. Over time, these issues have become increasingly important and are currently the driving force behind the efforts to find innovative solutions in many industrial sectors. The transportation sector, which is responsible for around 20 % of the share of annual greenhouse gas emissions [4], is affected directly. Light vehicles alone are responsible for 10 % of GHG (Greenhouse Gases) emissions and energy consumption. In general, a major portion of the related environmental impact occurs during its use phase. Fuel consumption (in the case of thermal powertrains), or energy consumption (in the case of full electric vehicles) are heavily influenced by vehicle mass directly.

As the number of road vehicles is rapidly increasing (from 700 million to 2 billion over the period 2000–2050 according to Refs. [5,6]), emission regulations are becoming increasingly stringent. In the European context, the path towards the complete decarbonization of the transport sector by 2050 is well defined. Progressive anti-pollution regulations have been adopted over the years [[7], [8], [9], [10]]. CO_2_ emissions of cars and LCVs (Light Commercial Vehicles) registered from 2025 onwards must be reduced by 15 %; from 2035 onwards such a reduction is increased to 37.5 % for cars and to 31 % for LCVs. The environmental problem affecting vehicles with thermal powertrains does not only include the global warming effect of CO_2_, but also the emission of large quantities of unburnt fuel and nitrogen oxides (NOx) [11].

**Fig. 1:**
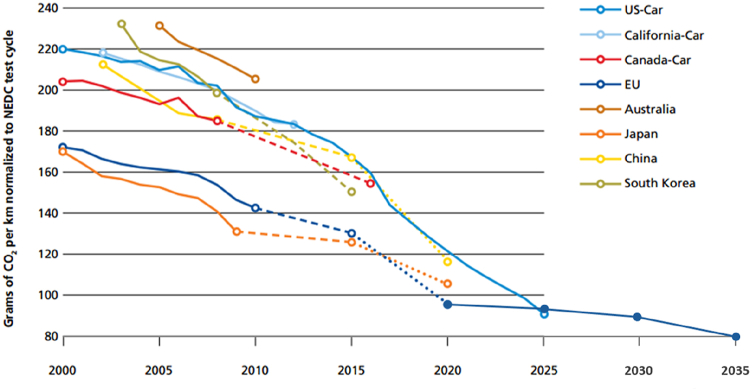
Evolution of CO_2_ emission limits for cars [119].

Fig. 1 shows the evolution of the maximum allowed CO2 emission limits and how these are becoming lower and lower as time goes by with the passing of new anti-pollution directives.

Corrective actions are therefore necessary to guide the automotive and transportation sectors in general towards more sustainable solutions capable of limiting the environmental, economic, and especially social costs linked to air and environmental pollution. The problem described above can be approached by improving the energy efficiency of the vehicle fleet and through the introduction of alternative powertrains.

One solution is to shift the circulating fleet of internal combustion engine vehicles towards the use of biofuels and synthetic fuels. Now, the largest investments are directed towards the development of BEVs (battery electric vehicles), i.e. purely electric vehicles. Currently, purely electric vehicles are still expensive and have certain issues that can make them unattractive to the consumer. These certainly include the limited range, the duration of the recharging process together with the poor availability of charging stations, the degradation of the battery pack over time and the impact of outside temperature on vehicle performance and mileage range. The overall impact in terms of CO_2_ for the car as a whole remains also related to the energy mix of the countries where it is produced and then used and disposed of. One of the other aspects that must be taken into account is the social and environmental impact of the ever-increasing demand for minerals and rare earth elements needed to manufacture batteries.

To overcome these problems, it may be interesting to consider hybrid solutions (with internal combustion engine or fuel cells), or to extend the range by means of appropriate strategies, such as the optimization of energy management on board the vehicle (for example through ad hoc logics of regenerative braking [12], to improve energy efficiency, or, indeed, through strategies to lighten the vehicle.

Over the years, several proposals have been made to improve the energy efficiency of vehicles and reduce the use phase impact. The following are some of the most relevant examples found in scientific literature.

• Development of improved next-generation powertrains with higher efficiency: high-efficiency ICE (Internal Combustion Engine), hybrid or electric [[13], [14], [15], [16]], and innovative power management strategies [17].
• Adoption of alternative energy sources to fossil fuels such as biofuels [18].
• Implementation of electronic speed controls to reduce energy and fuel consumption by making the powertrain work in the areas where its efficiency is greater [19].
• Improved aerodynamics through optimization of body shapes [20].
• Reduction of the vehicle mass (lightweighting) [[20], [21], [22], [23], [24]].

Among the above points, lightweighting is considered a very effective strategy for improving the overall efficiency of vehicles [23,[25], [26], [27], [28]]. This is valid both for vehicles with a traditional powertrain and for full electric vehicles. The latter ones, given the important masses involved mainly due to the battery pack, would benefit most from weight reduction as it would also enable the adoption of larger batteries, hence also improving the range which, nowadays, is their greatest limitation. It should also be stated that, as a secondary effect, lightweighting would also allow to downsize of the powertrain and transmission systems, at least to a certain extent.

Unfortunately, the current trend is towards a steady increase in terms of passenger car weight. The main reasons for this are given below.

### 1.1 Active and passive safety

The safety of vehicle occupants is an extremely important concern during the design phase of a vehicle. The chassis is designed to withstand the impacts related to crash tests, that have become increasingly more demanding over time. These passive safety requirements have led car manufacturers to strengthen the chassis in various areas such as the A and B-pillars, causing an increase in the overall mass. The various airbags located around the cabin also contribute to such an increase.

The development of security standards also requires the installation of additional devices such as actuators, cameras, and radars for driver assistance (ADAS). When analyzing these systems, it is necessary to consider not only the increase in mass due to the individual device, but also that of the electronics required for its management and control with the associated control units and cables. Sensors and control units must be connected to each other by wiring harnesses (often redundant for safety and electrical noise suppression reasons), which currently impact about 70 kg of the overall vehicle mass [29]. While a traditional vehicle features about a hundred sensors [29], this number grows to six times as many for electric vehicles and will be ever greater in the future when self-driving vehicles will be introduced. It is therefore intuitive to think that the increase in on-board electrical devices causes the overall mass of the car to rise; this increment in mass is even more pronounced in the case of electric vehicles, featuring complex electronics and the presence of the battery pack.

### 1.2 Emissions

Increasingly demanding requirements on emissions reduction have led to the introduction of complex and heavy anti-pollution systems such as catalytic converters, particulate filters and AdBlue systems in diesel-powered cars. These devices, which are mandatory for homologation, inevitably increase the weight of the car.

### 1.3 Comfort

The ever-increasing pursuit of occupant comfort requires the introduction of additional mass such as sound-absorbing materials inside door panels and chassis voids together with insulating windscreen and side windows to reduce resonance effects and perceived noise on board. This category also includes all the additional options demanded by the market, such as electronically controlled gearbox, all-wheel-drive transmission, rear-wheel-steering, electric adjustable seats, infotainment systems, dedicated air conditioning for each seat, GPS navigation, various other electric actuators etc, also adding weight.

### 1.4 Market demand

**Fig. 2:**
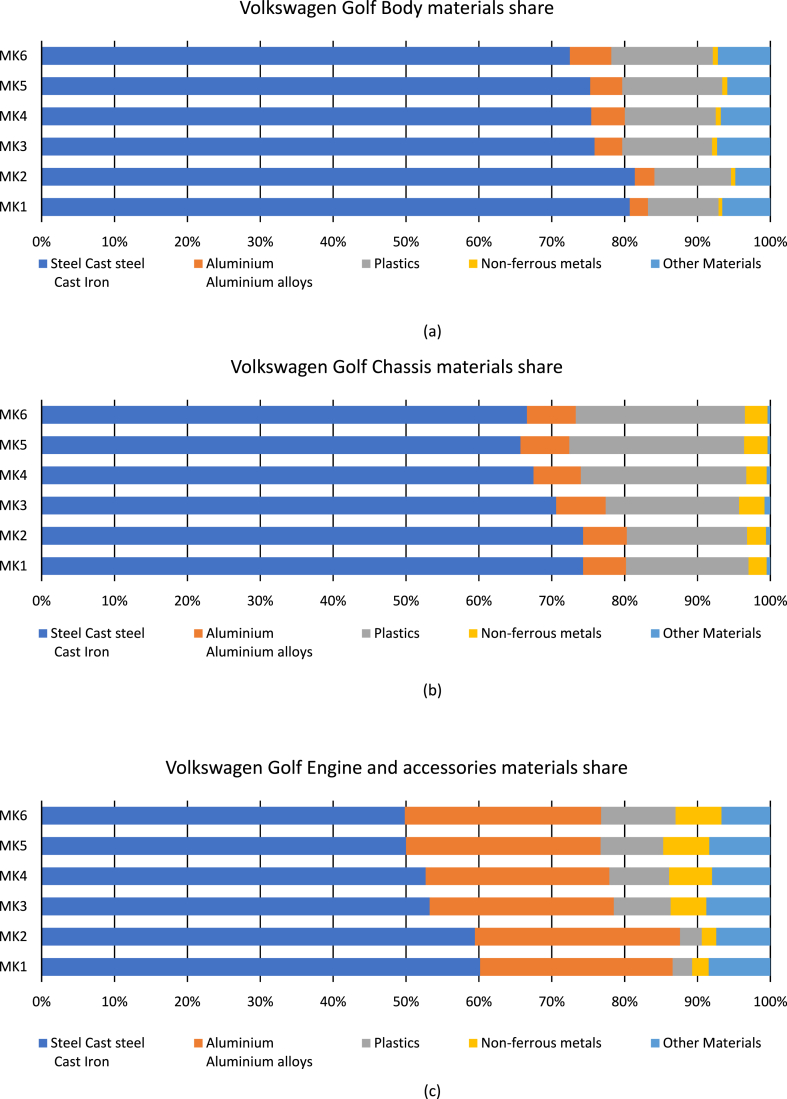
(a) body materials share, (b) chassis materials share, (c) engine and accessories materials share.

The last reason is also dictated by the market, which keeps shifting from the sedan and small cars more and more towards heavier and bulkier SUVs and crossovers. Over the three decades analyzed by the report [30], the average European car has increased in size by 10 cm in length, 4 cm in width and 2 cm in height. The confirmation of this trend is also found in the detailed LCA (Life Cycle Assessment) analysis of the first 6 Volkswagen Golf generations from 1976 to 2012 [31]. In addition to the increase in weight from 830 kg to 1215 kg corresponding to 46 % there is also a change in the materials that make the car up. The amount of steel and cast iron has decreased and has been replaced by alternative materials with lower density such as plastics and nonferrous metals (for more details see Fig. 2).

Now going back to lightweighting itself, the way in which can be carried out are varied, with the main ones listed below.

Lightweighting can be accomplished through material replacement by opting for alternatives with lower density and optimized design [13,22,[32], [33], [34], [35]]. A reference example is the use of tailored blank steel sheets which are a category of semi-finished products consisting mainly of sheets characterized by local variation in thickness, material, or coating. The use of these sheets, in the body frame construction, makes it possible to selectively toughen only those areas where it is necessary while containing the weight of the finished product as a whole [36]. Research is very active also in innovative materials such as advanced high stress steels (AHSS), i.e. steels with improved mechanical properties. The use of these solutions in chassis construction can make it possible to reduce the sheet metal thickness without any loss of strength and stiffness [37].

In the specific case of electric cars, most of the weight resides in the battery pack; the introduction of devices such as fuel cells [38] or hybrid powertrains, despite the increase in complexity, would make it possible to reduce the capacity, thus the size and weight, of the battery pack without affecting the range.

The implementation of optimized regenerative braking logics [39] would allow for more efficient recovery of kinetic energy and the possibility of installing smaller battery, thereby reducing the overall mass of the car.

Improving and developing batteries by introducing cells with higher energy density. This would reduce the size and the mass of the battery pack, this goal being the focus of worldwide research activities of vehicle and battery manufacturers [40].

In recent years, the market share of vehicles equipped with hybrid and full electric powertrains has been slowly increasing [3]. These innovative technologies ensure the reduction of greenhouse gases and other emissions at least locally, therefore significantly improving air quality in metropolitan areas where the problem is most impactful.

The test case analyzed in Ref. [41], and [42] considers a light commercial vehicle (LCV) (EU Category N1, Class III) equipped with a diesel engine subjected to a 20 % lightweighting. The results obtained provide a reduction from 1 % to 5 % in NOx emissions and from 1 % to 7 % in PM10 emissions. These values, compared to the best-case scenarios, reach up to 20 % in the case of NOx and 50 % for PM10, demonstrating, in fact, that lightweighting is a very valid measure to approach the problem. On top of that, the reduction of pollutants, although small, has a non-negligible effect on the social cost as these substances are the main responsible for respiratory diseases.

The present work aims to analyze the scientific literature concerning lightweighting in the automotive sector to define the state of the art.

The effects of vehicle lightweighting can be assessed from different points of view such as energy efficiency, material use, vehicle design and dynamics. For the sake of simplicity, only the first area will be dealt with, the effects of which can have an immediate impact on energy savings. The study of materials, design techniques and, above all, the impact on vehicle dynamics and active safety will be dealt with in a future work to give them the attention they deserve.

This paper is organized as follows:

• Section 2 contains the materials and methods used in writing this paper. This part also includes the bibliographic search method and the criteria for selecting and excluding scientific articles.
• Section 3 contains a description of the types of models for the simulation of longitudinal vehicle dynamics and the analysis of energy effects. The LCA approach for complete vehicle life cycle analysis is also mentioned.
• Section 4 finally presents the conclusions which are drawn from the analysis of the scientific literature.

## 2 Materials and methods

The literature review process began using the Scopus search engine, entering the keyword “lightweighting” (to be searched in the article title, abstract and keywords) and analysing all the titles identified starting from 2018, in order to obtain the most recent papers, up to November 9, 2022, the date on which the first phase of bibliographic research began.

Initially, numerous scientific articles were discarded based on the title, as they were not considered significant for the review work covered by this paper. Subsequently, additional articles were excluded after a thorough review of the abstracts, and ultimately, further ones were omitted based on a careful analysis of the entire articles. From this first search, based on title and abstract, the following articles were identified [[43], [44], [45], [46], [47], [48], [49], [50], [51]].

Following a careful reading of the entire text, articles [[45], [46], [47], [48], [49]] were subsequently discarded.

In particular [45], considers different alloy and technologies for components manufacturing with the aim of lightweighting, considering the transition from internal combustion engine to electric vehicles. It declares that the aim of lightweight design is to build structures with a minimal use of materials and an optimized utilization of the material strength. This work is mainly focused on materials; therefore, it goes beyond the main topic of this study.

[46] is a review article concerning the comparison, based on LCA methodology, of two alternative strategies: lightweight materials and alternative powertrain selection. The first aspect concerns materials, the second is not directly associated with the theme of lightweighting. This article was therefore discarded.

[47]also deals with the two topics of the previous article, therefore it was discarded for the same reasons. Furthermore [47], focuses on the Indian fleet and was therefore considered too restrictive compared to the more generic review work that is to be achieved.

[48,49,49]were also discarded as they are closely related to the topic of materials, in particular the first considers the use of plastic to lighten electric and hybrid vehicles and the second instead reports a study in which predominantly metallic materials are considered (aluminium, iron, steel, copper) regarding the mass of the various vehicle component.

From this first bibliographic search, only four scientific articles considered of extreme interest were identified. For this reason they were carefully analyzed, and then appropriately integrated with the information useful for the revision.

Two of these papers feature Del Pero as first author [50,51], it was therefore decided to delve deeper by searching for useful articles on his author profile in Scopus together with his co-authors, identifying further seven articles to be included in the review: [20,[52], [53], [54], [55], [56], [57], [58]].

Other relevant articles and documents were found by using the keywords “lightweighting”, “Light-Weighting” (for example, [24]), “forward-facing backward-facing model” [59], “driving cycles” [60] and “Life Cycle Assessment” (for example, [61,[61], [61], [62], [63], [64], [65], [66]]) within the Scopus search engine (searching in the title, abstract and keywords), and adding any references of selected papers as well.

Other articles were included in the study thanks to the previous experience of the research team, which has already worked on this area [[67], [68], [69], [70], [71], [72], [73]].

In general, as an inclusion criterion it was decided to include in the review work those articles that dealt with lightweighting, in the automotive field, with particular reference or with useful references to the topic of reducing consumption. Instead, it was decided to discard from the study those papers that do not refer to the reduction of consumption and which, for example, focus almost exclusively on the choice of materials and shape geometry to achieve vehicle lightweighting.

In Table 1 we provide a summary of the scientific literature that was reviewed in which the exclusion criteria are reported.

## 3 Longitudinal dynamics simulation models

Fuel and/or energy consumption is directly influenced by vehicle mass: article [74] shows that around one third of total fuel consumption on a given mission is related to displacing the vehicle mass. The resistance forces acting on a vehicle in motion are the following:

• **Inertia:**

(1) $$\vec{F_{i}}=m\vec{a}$$

• **Grade resistance:**

(2) $$F_{g}=m\vec{g}\sin\left(\alpha \right)$$

• **Aerodynamic resistance:**

(3) $$F_{aero}=1/2*\rho C_{x}A_{f}v^{2}$$

• **Rolling resistance:**

(4) $$F_{roll}=\left(a+bv+cv^{4}\right)mg$$

In equation (4), the coefficients usually have the following values a=0.01÷0.025, $b=2.5∙10^{-5}$ and $c=3.5∙10^{-10}$ [75].

From the previous equations, it can easily be seen that the main sources of resistance depend on the term of mass m directly. It should be noted that the first two contributions, inertia in particular, are partially recoverable through additional systems such as regenerative braking; this effect can be exploited only in hybrid and electric vehicles by using the electric traction motor as a generator. Regenerative braking increases the overall energy efficiency of the vehicle and justifies the development of hybrid powertrains. Reducing the overall vehicle mass reduces in turn the contribution of resistant forces thus the energy required for driving a vehicle from A to B.

Aerodynamic and rolling resistance are completely dissipative sources, consequently the only effective countermeasure to improve energy efficiency is to reduce their contribution. Aerodynamic resistance depends mainly on the square of speed. In its formulation however it is also possible to find an indirect relation with mass through the term $A_{f}$, which is the frontal area of the vehicle: as a matter of fact it is intuitive to think that large vehicles have a large frontal area. Rolling resistance is due to the interaction phenomena between the ground and the tires. It is generated through two mechanisms: tire hysteresis, and plastic deformation of the asphalt. The first is typical of viscoelastic components where the energy spent during the deformation phase is greater than that returned in the recovery phase. During rolling, this exchange occurs cyclically, and part of the energy is dissipated, causing an increase in temperature.

To evaluate the effectiveness of lightweighting, it is useful to develop meaningful criteria for testing procedures along with metrics for the aggregated evaluation of energy savings.

The following sections are aimed at presenting the state of the art methods for the evaluation of automotive lightweighting strategies with the main focus on energy efficiency.

Below, the following aspects of the problem will be discussed in detail:

• Analysis of the literature concerning simulation models for longitudinal dynamics.
• Analysis of the driving cycles required for energy estimation.
• Analysis of the energy effects of lightweighting and how the results can be evaluated.

Since the evaluation of the effects related to mass reduction through testing campaigns with a real vehicle would be expensive in terms of money and time, appropriate simulation tools should be developed to estimate the energy consumption of the vehicle. At this stage it is therefore necessary to develop calculation models capable of simulating the longitudinal dynamics of the vehicle. The current reference standard for simulation is the MATLAB-Simulink software environment [76], which allows for rapid and intuitive development of complete simulation models where various functionalities with modular logic can be integrated.

Simulation models require the following elements:

• **Virtual driver model:** This function is usually performed by a PI (Proportional-Integral) controller aimed at following the desired speed profile through acceleration and braking.
• **Driveline model:** I.e., a model of the gearbox with its inertia, brakes, tires, and any differentials.
• **Powertrain model:** generally, with a torque curve as function of engine speed and efficiency maps based on throttle percentage. The modular approach allows the easy integration of different powertrain options, thermal, hybrid, electric, fuel cell, etc.
• **Energy management model:** this element contains the battery, the inverter, the BMS (Battery Management System), the charging circuit and all the control electronics required to manage the energy flows exchanged between the components.
• **A mission profile:** defined by means of a time history in terms of speed, eventually coupled with elevation and payload variation.

The simulation models proposed in the literature can be distinguished into forward-facing and backward-facing models [59] depending on the direction of the computational flow of the variables involved. In addition to these two types, there are hybrid models that can combine the advantages of both calculation modes.

• **Forward-facing models**

**Fig. 3:**
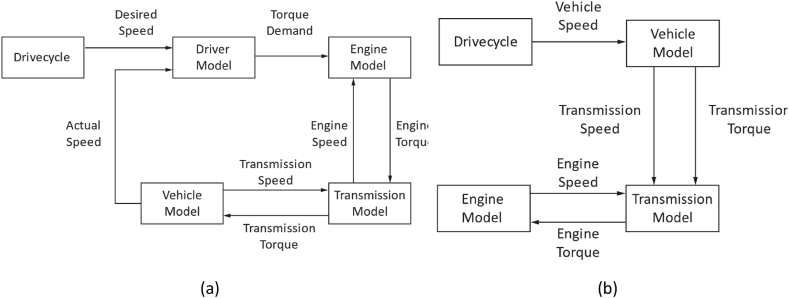
(a) Forward-facing calculation diagram.; (b) Backward-facing calculation diagram.

Forward-facing simulation models (Fig. 3a), such as the one proposed in Ref. [72], are based on a virtual driver model, usually a PI controller, activating throttle and brake inputs to follow the speed profile provided as a target. The computational flow of torques, forces, speeds and so on goes from the motor to the wheels. In this calculation model, the causal relationship between driver and driving cycle is maintained, and it is possible to directly account for torque and power limitations if the vehicle is unable to chase the speed target. Due to the PI controller, this approach entails a large computational burden that penalizes the performance of the simulation model.

• **Backward-facing models**

In backward-facing models (Fig. 3b), the driving cycle is rigidly imposed and used backwards for the calculation of the torque. The computational flow goes from the target speed profile to the wheels, then to the motor and battery pack. In this case, the causal relationship between the driver and the driving cycle is not maintained. These types of simulation models assume that the powertrain has no limitations in terms of torque and power and that consequently the speed profile is always followed. This feature improves computational efficiency but requires dedicated controls to check for these limitations.

• **Hybrid calculation models**

Recently, innovative calculation models have been developed, such as the one described in Ref. [68]. They can combine the advantages of previous ones. These models improve efficiency by reducing the computational burden. By default, the backward-facing approach is used and at each instant the torque and power demands are compared with the constraints of the powertrain. If these restrictions are not met, the calculation model automatically switches to forward-facing mode.

The input required by all three types of models is the target speed/elevation profile to be followed by the vehicle during the simulation. In addition, it is necessary to provide a set of parameters defining the layout of the vehicle e.g. mass, aerodynamic coefficients, aerodynamic maps (if any), the parameters of the wheels and tires, the powertrain characteristics, possibly the parameters of the braking system etc. These parameters, and their completeness in particular, depend on the model considered and on the desired precision and accuracy, for example a more sophisticated model can consider an aerodynamic map, while a simplified one can consider a constant drag coefficient along the whole simulation. Some models can consider the Pacejka coefficients of the tires, while others consider a simple rolling resistance coefficient. More accurate models can use the moments of inertia of the various rotating components such as transmission and wheels, tire slippage, limitations given by the BMS (Battery Management System), etc. However, the greater the accuracy of the model and the greater its computational burden; it is therefore necessary to find a suitable compromise between simulation speed and accuracy.

About model validation, it can take place by comparing the simulation results with experimental data obtained on the real vehicle, or by means of a comparison with the results of an already validated model [68].

The pros and cons of the calculation models described have been collected in Table 2.

**Table 1:** Summary of the selected scientific articles.

Reference	Inclusion criteria	Exclusion criteria	Search keyword(s)	Used?
[20]	Relevance to the topic	/	Lightweighting	Yes
[24]	Relevance to the topic	/	Light-weighting	Yes
[43,44]	Relevance to the topic	/	Lightweighting	Yes
[45,46];	/	Dealing with materials and LCA	Lightweighting	No
[47]	/	Studies conducted on a specific geographical area	Lightweighting	No
[48,49]	/	Dealing with materials only	Lightweighting	No
[50,51]	/	Relevance to the topic	Lightweighting	Yes
[[52], [53], [54], [55], [56], [57], [58]]	/	Relevance to the topic	Lightweighting	Yes
[59]	/	Relevance to the topic	Forward-facing backward-facing model	Yes
[60]	/	Relevance to the topic	Driving Cycles	Yes
[61,[61], [61], [62], [63], [64], [65], [66]]	/	Dealing with materials and LCA	Life Cycle Assessment	No
[[67], [68], [69], [70], [71], [72], [73]]	/	Relevance to the topic	Lightweighting	Yes

**Table 2:**
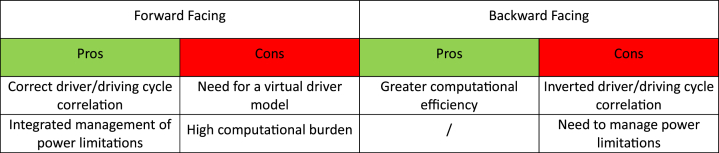
Pros and cons of forward-facing and backward-facing calculation models.

The main feature observed in the simulation models is modularity, which allows them to be easily adapted to different types of vehicles and powertrains, thus providing flexibility of use. The model described in Ref. [68] adapts easily to vehicles with electric or hybrid powertrains and to applications with auxiliary generators as range extenders [71,73], fuel cells [77] and regenerative braking system [39,78].

### 3.1 Driving cycles

To work properly, the simulation models require a so-called driving cycle as input. This represents a certain vehicle “mission” provided as a time-history of speed and elevation. Driving cycles are usually provided by the reference standards or can be obtained through the acquisition of data from real vehicles.

The most frequently used driving cycles for testing and validating results are the following ones.

• **NEDC (New European Driving Cycle)** [79]

**Fig. 4:**
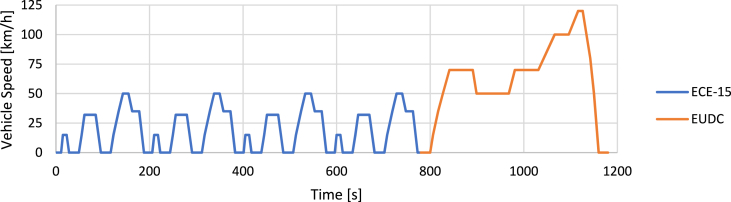
NEDC driving cycle.

The NEDC driving cycle (Fig. 4) was intended to represent typical car use on the European territory to assess emissions and fuel consumption. This cycle consists of an urban section represented by repeating the ECE-15 cycle four times plus an extra-urban EUDC section until a speed of 120 km/h is reached.

Currently, this test method is no longer used and from 2018 has been replaced by the WLTP (Worldwide Harmonized Light-Duty Vehicles Test Procedure) testing methodology.

• **WLTC (Worldwide harmonized Light-duty vehicles Test Cycles)** [80]

**Fig. 5:**
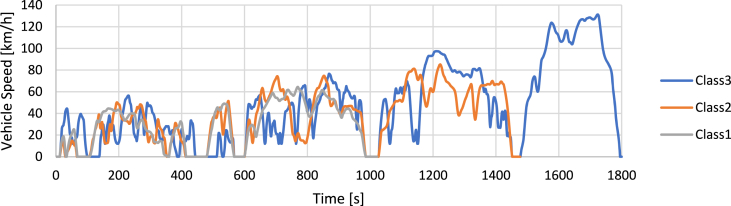
WLTC driving cycles.

The WLTC driving cycles (Fig. 5) are part of the harmonized WLTP testing procedure and are distinguished into different categories according to the power-to-weight ratio of the vehicle tested. This makes it possible to adapt the testing procedure to different vehicles while preserving typical driving cycle differences between the various classes.

• **CADC (Common Artemis Driving Cycles)** [81]

**Fig. 6:**
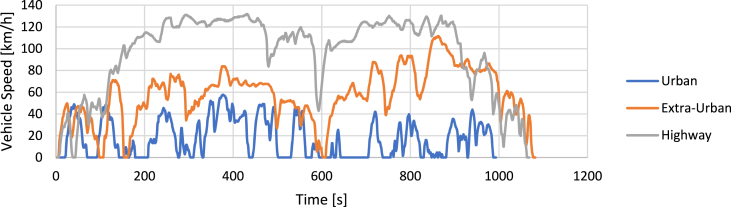
CADC driving cycles.

ARTEMIS-type driving cycles (Fig. 6) are obtained through statistical analysis of databases of real data acquisitions.

The main characteristics of the driving cycles have been collected in Table 2.

**Table 3:** Driving cycles data.

	NEDC			WLTC			CADC		
	ECE-15	EUDC	NEDC	Class 1	Class 2	Class 3	Urban	Extraurban	Highway
Duration [s]	195	400	1220	1022	1480	1800	993	1082	1068
Max Speed [km/h]	50	120	120	64.4	85.2	131.3	57.7	111.5	131.8
Avg Speed [km/h]	18.3	55.3	26.0	28.5	35.7	46.5	17.6	57.4	96.8
Max Acceleration [m/s^2^]	1.04	0.69	1.04	0.81	1.00	1.75	2.86	2.36	1.92
Max Deceleration [m/s^2^]	−0.97	−1.39	−1.39	−1.14	−1.17	−1.50	−3.14	−4.08	−3.36
Avg Acceleration [m/s^2^]	0.43	0.38	0.40	0.17	0.25	0.36	0.51	0.41	0.34

An analysis of the results of the simulations performed in Ref. [82] shows that the NEDC cycle is unsuitable for representing a real-life scenario of vehicle use due to excessively gentle accelerations (see Table 3). Consequently, the calculation model underestimates overall fuel consumption and emissions. In addition, it does not include any variant to consider the class of the vehicle being tested. WLTC and CADC speed profiles are more realistic by far because they are based on statistical processing of real-world acquisitions. Nevertheless, the computation of vehicle emissions is still considered as underestimated [82]. At present, even considering the limitations mentioned above, the reference driving cycle for Europe is the WLTC.

The driving cycles presented above are just a few among all those available [83]; the choice depends on the type of longitudinal behavior that the computational model wants to simulate. Some alternative examples are SFTP-US06 [84], JC08 [85], and HWFET [86] cycles. With the development of the electric car industry, it is also necessary to introduce driving cycles that consider the specific characteristics of these vehicles such as the possibility of slowing down using regenerative braking. The methodology proposed in article [52] is a suitable starting point for driving cycles in the urban environment. The example refers to the city of Florence, but the methodology remains viable and applicable to other places and hereafter replicable to a situation that can represent the specific scenario of the centre of a city.

**Fig. 7:**
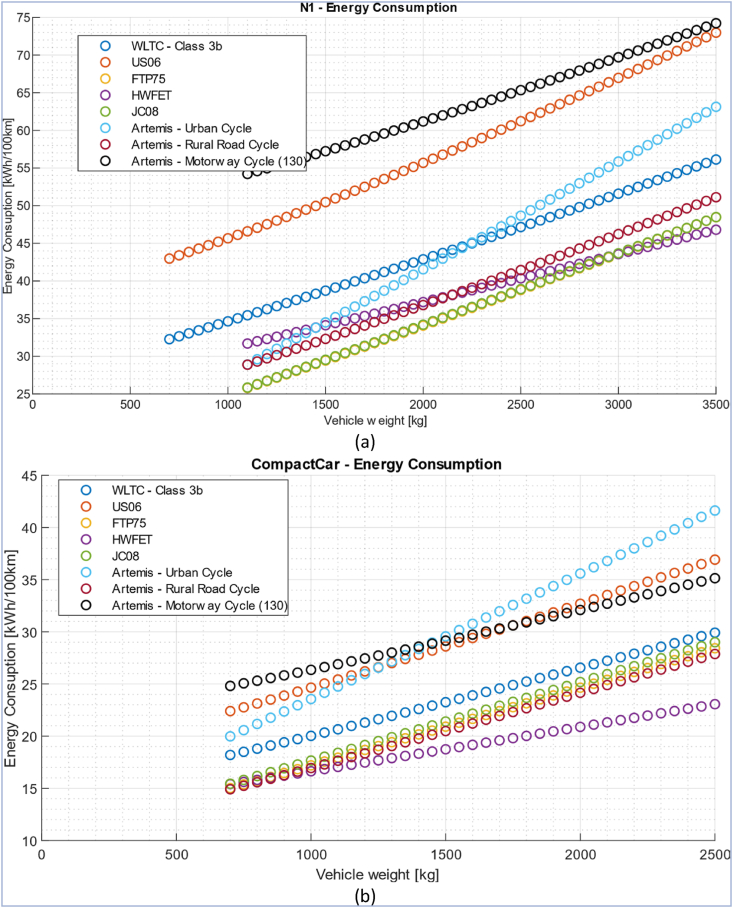
Average energy consumption, for (a) an N1 category vehicle and (b) a compact car, without regenerative braking, on the following standard driving cycles: WLTC (class 3b); US06; FTP75; HWFET; JC08; Artemis, Urban Cycles; Artemis, Rural Road Cycle; Artemis, Motorway Cycle (130) [67].

**Fig. 8:**
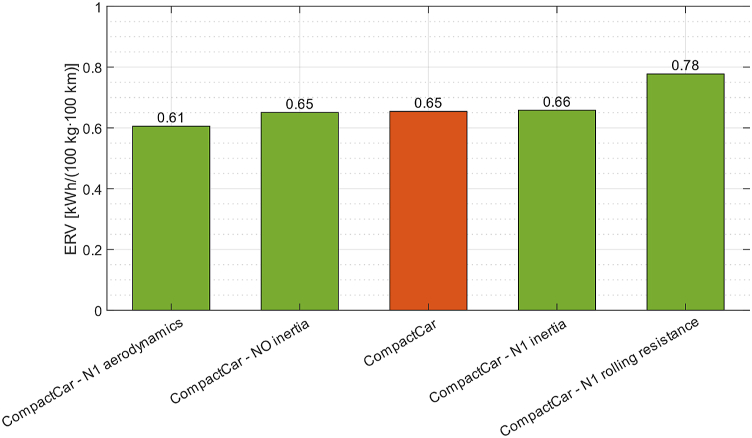
ERV values obtained for a compact car [67].

As can be seen from the graphs in Fig. 7, as the driving cycle changes, energy consumption varies, and the result obtained from the vehicle lightweighting process varies as well. For example Fig. 7 a shows how lightweighting of the compact car considered in study [67] provides greater benefits in terms of reducing consumption when associated with the Artemis urban driving cycle, rather than with the WLTC driving cycle. Furthermore, depending on the class of vehicle and its mass, one driving cycle may be more demanding than another or vice versa. In fact, as can be seen from Fig. 7, the Artemis motorway cycle is the most demanding in terms of energy among all the cycles considered for the compact car. The same would apply to N1 vehicles, but only below a 1400 kg vehicle mass, which is scarcely realistic. Above that figure the Artemis urban cycle is the most energy demanding one. The next section contains further information on this matter, regarding the ERV (Energy Reduction Value) index in particular, see Fig. 8.

### 3.2 Energetic effects

To assess the effects of lightweighting properly it becomes necessary to develop specific metrics to represent the results in an aggregated and intuitive way. Therefore, coefficients that quantify the energy or fuel savings as a function of the weight reduction applied and the mileage travelled are required.

The scientific literature analyzed [24,53,54,62,67,87,88] uses the following metrics:

• **ERV (Energy Reduction Value)** [67,88]

Defined as $ERV=\frac{\Delta EC}{\Delta m},\left[ERV\right]=\left[\frac{kWh}{100km\;100kg}\right]$, where ΔEC and Δm are the variations compared to the reference case in terms of energy consumption and mass respectively. Physically this coefficient represents the reduction in energy required to complete the driving cycle. The coefficient is normalized for a 100 kg mass reduction and a distance of 100 km. From the simulations carried out in Ref. [88], the value of ERV ranges from 0.47 up to 1.17 depending on the class of vehicle considered. In general, the ERV coefficient increases as the vehicle size class increases. The ERV metric is particularly suitable for describing energy savings for electric vehicles because it is a direct measure of energy.

• **FRV (Fuel Reduction Value)** [53,54,62,87,88]

Defined as $\left[FRV\right]=\frac{\Delta FC}{\Delta m},\left[\frac{l}{100km\;100kg}\right]$, where ΔFC and Δm are the variations compared to the reference case in terms of fuel consumption and mass respectively. This coefficient indicates the amount in liters of fuel, gasoline, or diesel, saved for at 100 kg weight reduction along a 100 km distance. This metric is used extensively in the articles [53,54] and depends mainly on the class of vehicle and the driving cycle used during the analysis. This index is particularly appropriate for the evaluation of cars with ICE powertrains because it provides a metric that is easy to understand without the need for conversion. FRV for gasoline cars ranges from 0.159 to 0.237 for primary lightweighting and between 0.252 and 0.477 when secondary effects such as powertrain, and transmission resizing are also evaluated. For diesel cars, the values obtained are between 0.115 and 0.143 for primary lightweighting and within 0.142 and 0.388 for secondary effects [53,54].

• **IRV (Impact Reduction Value)** [88]

This index is defined as $IRV=ERV∙GWP_{kWh},\left[IRV\right]=\left[\frac{kgCO2_{eq}}{100km\;100kg}\right]$, and physically represents the mass of $CO_{2eq}$ saved through a mass reduction of 100 kg along a 100 km distance. This index, with the $GWP_{kWh}$ coefficient, quantifies $CO_{2}$ emissions for every kWh of energy produced. This coefficient depends on the type of power mix used to produce the energy. This metric also makes it possible to consider the environmental impact that various energy production technologies have. Intuitively, this value will be higher the greater the share of fossil fuels used in the energy mix is. The IRV values estimated range between 0.02 and 1.10 [88].

Article [67] proposes a detailed study carried out through sensitivity analysis on the parameters that can most influence fuel consumption. What emerges from the work is that the contribution of vehicle inertia can be neglected, but special attention should be paid to the modeling of aerodynamics and rolling resistance as they significantly change the consumption behavior of the vehicle. Considering instead the phenomenon from the point of view of the ERV coefficient, it is observed that this value increases as vehicle mass increases because the potential contribution of lightweighting is greater. The effects of aerodynamics on ERV are small and can therefore be neglected. Rolling resistance, on the other hand, greatly changes the ERV since it is directly dependent on mass.

Fig. 8 shows a set of results obtained from the simulations performed in Ref. [67]. The histogram shows in orange the ERV value for the reference case of a compact car and in green the case studies related to the sensitivity analysis. The effect of driveline inertia is practically negligible and there is no difference between the extreme cases "CompactCar - No inertia" and "CompactCar - N1 inertia" even though in this case the rotational elements inertia value related to a higher-class vehicle was deliberately used to assess the sensitivity of the model to this parameter. As anticipated, the parameters that affect ERV the most are the aerodynamic configuration and most of all the rolling resistance.

**Fig. 9:**
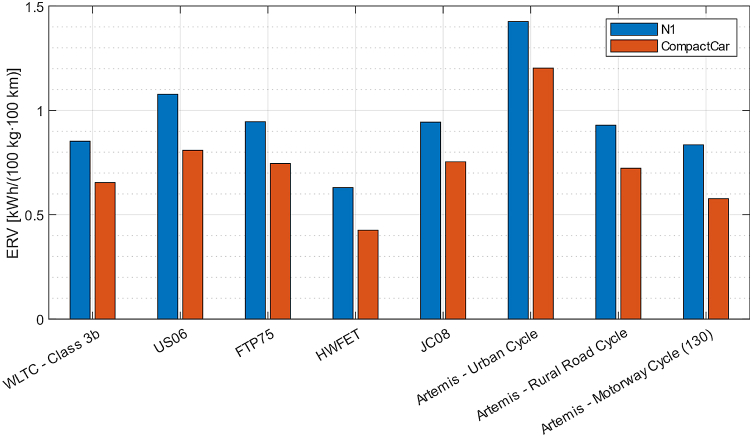
ERV index, for different standard driving cycles, for the N1 vehicle and for the compact car [67].

The study [67] also reveals that the ERV index varies depending on the driving cycle considered and on the vehicle under examination, as can be seen from Fig. 9. In particular, although with different ERV index, both vehicles under study benefit more from the effects of lightweighting when on the Artemis urban cycle, for which the ERV index is higher than all the other cycles examined, while the ERV index is the lowest for both vehicles on the HWFET cycle.

### 3.3 LCA analysis

The Life Cycle Assessment (LCA) approach, allowing for the entire life cycle of the vehicle to be considered, is used to carry out full assessment of its environmental impact. This method of analysis is regulated by ISO 14040 and 14044 standards [89,90], defining the principles and methods for assessing the environmental impact of a product from the extraction of the materials needed for its production all the way down to the recycling stage. For the transport field there are many examples of LCA in the literature [[55], [56], [57],^61^,^63^]: interest in this is growing, especially in the automotive sector [63,64,[91], [92], [93], [94], [95]]. Through this method, it is therefore possible to address the problem with a broader perspective considering the entire life cycle of the product and consequently leading the industry choices toward more sustainable solutions. For example, it has been found that the use of lightweight materials such as magnesium alloys or carbon fiber, while providing good opportunities for lightweighting, requires a large amount of energy in both the production and recycling phases, partially cancelling out the benefits obtained in the use phase [58,65,[96], [97], [98]].

The transition to electric traction also carries all the problems associated with the batteries, which are a major issue both in terms of the raw materials needed and the production processes that inevitably generate emissions and waste products. Regarding this, further analysis should be carried out to assess whether the emissions generated during the production and recycling phases of the battery pack cancel out the savings that are achieved during the vehicle's use phase. Article [99] applies the LCA approach to the life cycle of some families of batteries for electric traction differing in chemical composition. What is noted is that the main contribution to the environmental impact lies in the preliminary stage of preparation of the materials needed to build the batteries [66]. In conclusion, the production phase is the most impactful in terms of resource demand by far. In general, the manufacturing process of large batteries impacts the environment in several ways, the main ones being water, metal, and energy consumption, particulate emission, and global warming. Social impact is also mentioned sometimes [100].

When evaluating lightweighting operations the secondary effects induced should also be considered. In general, reducing vehicle mass reduces the contribution of resisting forces and consequently energy/fuel consumption. Often the improved performance of the lightweighted vehicle compared to the reference case is not considered. During the development of a vehicle, its class, and consequently its expected performance, are fixed early in the design process. To keep the performance parameters unchanged, powertrain and transmission downsizing could be applied. Through this operation it is possible to further increase the energy savings indices. As reported in Ref. [74] the operations of adjusting the transmission and displacement ratios allow to go from an initial FRV of 0.15 up to the maximum of 0.39 and 0.45 respectively. In the case of diesel vehicles, there is an increase from 0.12 up to 0.30 and 0.29 in the scenarios described.

## 4 Discussion

The purpose of this paper is the analysis of the scientific literature regarding the effects of vehicle lightweighting from the energy point of view. It was then possible to reconstruct the analysis methodology allowing to obtain information on the effects of automotive lightweighting on energy consumption.

In this paper, unlike in those already available in the literature [101,102], lightweighting is not analyzed from the perspective of innovative materials [[103], [104], [105], [106], [107], [108], [109], [110], [111]], structural solutions [[112], [113], [114]], and manufacturing technologies [[115], [116], [117]] to make lighter components and cars. The focus of this review is to describe the state of the art on vehicle lightweighting by focusing on the beneficial effects that mass reduction can provide for energy efficiency, fuel consumption, and vehicle range. Cars equipped with an internal combustion engine would benefit from reduced greenhouse gas emissions and pollutants, while for full electric vehicles the main issue to be addressed is the improvement of range, which, to date, is their most limiting aspect. From a practical point of view the study, working methodology and simulation tools presented are useful in the vehicle development phase and can therefore be elements of support for the decisions that occur during the design phase of future cars to be released on the market.

It was possible to draw the following conclusions:

• Lightweighting can be a viable way to reduce energy consumption and, consequently, vehicle emissions.
• It is convenient to evaluate the energy effects of lightweighting through a model-based approach with computational models for longitudinal dynamics, limiting tests in the real environment only to those strictly necessary to validate the simulation models.
• In literature there are synthetic indices for the evaluation of lightweighting effects such as ERV or FRV. Their values strongly depend on the class of vehicle analyzed and the driving cycle used. Regarding this, it would be useful to define and unify specific testing procedures to derive this information and build a database to support powertrain design of next-generation vehicles.
• By considering the secondary effects of lightweighting through powertrain and transmission resizing, energy efficiency levels can be further increased.
• In the scientific literature reviewed, there are no studies of the effects of lightweighting on vehicle dynamics. This aspect holds considerable importance as it directly affects stability, handling, road holding and braking distances, which are vital for active safety, a fundamental aspect that is a priority in the design phase of a vehicle. The possibility of combining improved energy efficiency, reduced emissions, and increased active safety in the vehicles for everyday use would ensure a great advancement in the automotive sector and therefore justifies the interest of scientific research in this regard.
• Lighter vehicles would also carry overall improvements in terms of passive safety (as kinetic energy to be dissipated in a crash is directly proportional to vehicle mass) as well as in terms of impact on the road infrastructure. This aspect, however, is not dealt with in this paper.

The study and the method of work that have been presented are not free of limitations. It should always be kept in mind that the simulation models presented, although validated, are a simplification of reality and therefore may lead to partially inaccurate results and considerations. As already stated, a key issue that seems to be little addressed in the relevant scientific literature is that of the effect of vehicle mass variation on vehicle dynamics. While the effects on energy efficiency such as improved range and reduced fuel and energy consumption have been thoroughly analyzed [50,51,53,56,67,78,118], those affecting vehicle dynamics are not so intuitive. Reducing mass involves changing some significant vehicle parameters such as the inertia matrix, weight distribution, and height of the centre of gravity. These changes affect the behaviour of the car in terms of tire forces, stability, handling, road holding, and braking distances.

A specific investigation will be carried out in the near future on the beneficial effects that lightweighting can have on vehicle dynamics, driving behavior and active safety. The results will be presented in a dedicated paper.

One of the next tasks consists of creating a database of ERV values for vehicles that differ by class and for which, if possible, experimental validation will be carried out. In addition to the energetic effects, metrics will be defined to evaluate dynamic effects such as vehicle stability and aspects including active safety. Therefore, the information gathered in this work may be useful to continue the project that is currently being carried out at the University of Brescia and that has the goal of evaluating vehicle lightweighting obtained through different technologies.

## Funding

The project is financed by the European Union, see below.

## Data availability statement

The data presented in this study are available on request from the corresponding author. The data are not publicly available due to the University of Brescia privacy policy.

## CRediT authorship contribution statement

**Andrea Candela:** Writing – original draft, Formal analysis, Conceptualization. **Giulia Sandrini:** Writing – review & editing, Visualization, Validation, Supervision. **Marco Gadola:** Writing – review & editing, Visualization, Validation, Supervision, Project administration. **Daniel Chindamo:** Supervision. **Paolo Magri:** Visualization, Validation.

## Declaration of competing interest

The authors declare the following financial interests/personal relationships which may be considered as potential competing interests Andrea Candela reports financial support was provided by 10.13039/501100000780European Union.
